# Health Tract, No. 301

**Published:** 1867-08

**Authors:** 


HEALTH TRACT, No. 301.

---

### ACIDTIY.

Acidity of stomach always arises from that organ not being able to digest, to work up the food eaten, to extract the nutriment which it contains, hence two results: First, the food decays, that is rots, becomes sour and generates a sour gas, which is belched up, causing a burning or raw sensation, located apparently at the little hollow at the bottom of the neck, or in that vicenity. Sometimes an acid fluid is generated and is belched up, and so very sour occasionly as to take the skin off of some parts of the throat, mouth or lips. Second, the food not being properly worked up, does not give out its nourishment, the system is not fed, and consequently becomes weak, the circulation becomes feeble, the feet grow habitually cold; the person is easily chilled, and dreads going out of doors; is happiest when hugging the fire, and takes cold so easily that the expression is frequently used, "the least thing in the world gives me a cold." When such a condition is reached these colds are so frequently repeated that before one is cured another comes, and there is a perpetual cough which the most unintelligent know is the certain harbinger, the forerunner of consumption of the lungs.

When persons are troubled with indigestion, and one of its effects, acidity, the advice given in nearly all cases is to take something to correct the acidity, such as cream of tartar, soda, saleratus, ammonia, the ley of wood ashes, and other alkalies. These things correct the acidity, but the stomach gets no power of a better digestion, the effects as far as sensation is concerned are removed, but the system continues to be improperly nourished; the man grows thinner, and weaker; and with wasting of flesh and strength, there is diminished power of circulation; the person becomes chilly, colds are taken from slight causes and at diminishing intervals, and before he knows it he has an annoying, hacking cough, which too often ends in a wasting, fatal disease.

When acidity follows eating, it is always because there has been an error in the quantity or quality of the food eaten; the stomach could not manage it, could not perform the work imposed upon it. The true remedy is to eat less and less at each meal, until no acidity is perceptable, or to change the quality of the food; and in a short time the stomach, not being overtasked, gets time to rest, to recupurate, to get strong; then it digests more food and digests it better, with the inevitable result of a more vigorous constitution, more power of endurence, more strength of body and greater elasticity of mind, more happiness and a spirit and energy to grapple with life's duties, which makes existence a pleasure.

